# Samba Sow: supporting regional research for context-appropriate medicines

**DOI:** 10.2471/BLT.21.031221

**Published:** 2021-12-01

## Abstract

Samba Sow talks to Andréia Azevedo Soares about the need for regional approaches to medical product development, including treatments for mild and moderate COVID-19.

*Q: You are a medical doctor and an epidemiologist. How did you develop an interest in those disciplines?*

A: For me the two have always gone together. I realized my passion for medicine very early in life, even before secondary school, partly because I was surrounded by so much infectious disease. The first disease that really got my attention was measles, but there was also meningitis, leprosy and onchocerciasis. I saw so many people get sick as a young boy, so many people die. I was shocked by what those diseases could do to people, but I was fascinated too, and I started studying biology with great interest at secondary school. But my early education went beyond that. My mother worked at a leprosy centre and I would help her there, seeing a lot of leprosy patients. So, I went to medical school with all that experience behind me, but my interest in epidemiology as a discipline really developed when I was introduced to Doctor George Soula, an epidemiologist who was running a WHO-funded onchocerciasis treatment trial and also looking into ongoing measles outbreaks. He was the first person to take me out into the field and that was tremendously exciting for me. And I continue to feel that excitement, including with my work on the ANTICOV trial.

*Q: What is the ANTICOV trial and why is it important?*

A: The ANTICOV trial is a large multi-African-country clinical trial that is using an adaptive platform to investigate drug treatments with the potential to treat mild-to-moderate cases of COVID-19.

*Q: Why focus on mild-to-moderate cases of COVID-19?*

A: Until recently, attention has tended to focus on developing treatments for severe COVID-19 cases. While such trials clearly have their place, they tend to reflect conditions in countries with more robust health systems than those found in the African context. The core idea of the ANTICOV trial is to identify drugs or combinations of drugs that are effective in treating mild-to-moderate COVID-19, thereby preventing the development of more serious cases which can end in hospitalization, overwhelming fragile health systems.

*Q: You mentioned the trial’s adaptive platform. Can you explain what that means?*

A: As the name suggests, it is a trial platform that allows for adaptation depending on results. Because we don't know what's going to work, we need processes that allow us to make substitutions or additions or recombinations until we get the final, most effective and acceptable health product. The trial started in September 2020 with the anti-malaria drug hydroxychloroquine and the antiretrovirals lopinavir and ritonavir which were all suspended in December 2020 after WHO updated its treatment guidelines. The trial is now focused on a new potential treatment combining the anti-parasitic nitazoxanide and the corticosteroid ciclesonide.

*Q: The ANTICOV trial focuses on African countries. Was this a deliberate choice?*

A: Yes. The trial brings together a consortium of 26 partners which includes leading African research institutions and international health organizations, the whole thing coordinated by the Drugs for Neglected Diseases *initiative* (a research and development non-profit organization). I personally am very happy to have watched the trial become a reality because I consider it vital to take a regional approach in developing medicines as well as other medical products, including vaccines of course.

“[There is] growing demand for more African-led research and development.”

*Q: Why is this regional approach so important?*

A: Africa presents several specific challenges that have implications for the way in which infectious diseases, including of course zoonoses such as SARS-CoV-2 (severe acute respiratory syndrome coronavirus 2), emerge and are distributed. I am talking about climate and ecosystem challenges, but also socioeconomic and cultural challenges. There are also significant resource challenges in many African countries that impact health systems and have a bearing on the kind of medical products best suited for use in fighting COVID-19. Generally speaking, our capacity to handle severe cases is very limited and our systems overall lack resources. This is certainly the case in Mali, where I have some insight, having had the privilege of serving as Minister of Health here. We not only lack resources but are still using public health strategies drawn up when we achieved independence in 1958. And this despite the fact that there have been major demographic and sociocultural changes in the country, not to mention changes in the climate, as well as technological advances and innovations in practice that have transformed health-care delivery. The weakness of our system was exposed in the 2014 Ebola outbreak and, I am sorry to say, the same thing is happening with COVID-19. My hope is that the pandemic will encourage a rethinking of priorities and some reflection regarding the best ways to reform the health-care system. What we really need at this point is to strengthen our primary care capacity, our community-based health system, and our emergency operating centre system. We also need to fully implement disease surveillance in our communities to support prevention. In the short term, we need to recognize that developing treatments that can be delivered outside of the hospital or health facility context is vital. That's why ANTICOV is so important for us.

The second key reason for pursuing a regional approach is to answer growing demand for more African-led research and development. ANTICOV goes some way to dispelling the notion that we, as a region, have tended to sit and wait for leftovers when it comes to new medical products, tools and strategies, including strategies for emergency situation management. There is considerable support therefore for developing our own trials and trial protocols in a collaborative manner. It is not that we want to go it alone. There has to be collaboration with international research and development networks, especially in a pandemic, and we will share our findings as needed. Finally, and this relates to what I just said about the need for Africa-led research and development, conducting trials in Africa is vital to building trust in local communities and overcoming hesitancy and resistance regarding new products.

*Q: What kind of resistance have you encountered in local communities?*

A: All kinds. From general concerns about sampling and measurement – Why do you have to take my blood? Why do you have to take my weight? Why are you measuring my height? – to more specific concerns that vary according to the medical product being tested. When a trial only studies women or children, the men are sometimes suspicious and ask to participate in the trial on their behalf. In such cases, you have to explain the scientific basis for certain decisions. You just need to explain. There are also other concerns based on misinformation or what might be termed post-colonial distrust. People sometimes say, “You are bringing products from white people to be tested on us” or “You want to give us HIV (human immunodeficiency virus) to kill us”. You hear all kinds of things in the field.

“Conducting trials in Africa is vital to building trust.”

*Q: How do you overcome such attitudes in the ANTICOV trial?*

A: By talking with communities. Communication is key. With ANTICOV, even before the trial protocol had been finalized, we went out to targeted communities and community members to provide information and to listen to people’s concerns. We met with high-level leaders such as elected political representatives but also with local groups such as women’s associations or religious assemblies. We also spoke with traditional healers who play a very important role in Mali. They can be very powerful in terms of influencing public opinion, so you can never exclude them. And we have been absolutely transparent. That's how you build trust. We are absolutely honest about what we know and what we do not know. And we have maintained that dialogue with follow up. We also showed people that the trial protocol had been submitted for official review and once we had official approval, we went back to the communities with consent forms which we translated into the local languages most commonly used, either in audio or written form, so that people would know exactly what was being proposed. This is a significant challenge in Mali where, despite the fact that French is the official language and Bambara the most widely spoken, more than 80 languages are spoken, 13 of which have the status of a national language. As a result of this commitment to communication and trust-building, the trial is progressing fairly well. 

*Q: How important will effective, affordable COVID-19 treatments be to countries that have limited access to vaccines?*

A: Very important for the reasons I have talked about, but it is clear that access to vaccines remains absolutely vital – as is vaccine uptake. I mention this because there is considerable resistance to vaccination developing in Mali and it is impacting vaccine roll out. This is partly because COVID-19 has become so politicized and partly because anti-immunization movements are getting stronger. We are also struggling on the non-medical intervention side. Something like half of the population does not believe in the threat posed by SARS-CoV-2 and are unwilling to observe prevention measures. I am afraid to say it reminds me very much of attitudes that prevailed during the Ebola outbreak. So, what is really important to me at this point, and what I find myself talking about most, is the need for people to wear their mask and observe physical distancing and hygiene protocols. And if they can get vaccinated, they should do so.

*Q: What worries you most when you think about the health challenges ahead in your region?*

A: We still have very high rates of maternal and infant mortality, and infant malnutrition and very low levels of coverage of basic primary health care, including antenatal care for a mother, or routine consultations for a child. It can be challenging even to find someone to take your blood pressure or give you an eye check-up. Hopefully the pandemic will focus attention on these weaknesses. We need to stop thinking about health expenditure as a cost and recognize that it is in fact an investment.

